# A Risk Scoring System Utilizing Machine Learning Methods for Hepatotoxicity Prediction One Year After the Initiation of Tyrosine Kinase Inhibitors

**DOI:** 10.3389/fonc.2022.790343

**Published:** 2022-03-08

**Authors:** Ji Min Han, Jeong Yee, Soyeon Cho, Min Kyoung Kim, Jin Young Moon, Dasom Jung, Jung Sun Kim, Hye Sun Gwak

**Affiliations:** ^1^ College of Pharmacy, Chungbuk National University, Cheongju-si, South Korea; ^2^ College of Pharmacy and Graduate School of Pharmaceutical Sciences, Ewha Womans University, Seoul, South Korea; ^3^ Department of Pharmacy, Asan Medical Center, Seoul, South Korea; ^4^ Graduate School of Converging Clinical and Public Health, Ewha Womans University, Seoul, South Korea; ^5^ Department of Pharmacy, Seoul National University Hospital, Seoul, South Korea; ^6^ Department of Pharmacy, National Cancer Center, Goyang-si, South Korea

**Keywords:** tyrosine kinase inhibitor, hepatotoxicity, prediction, machine learning, risk scoring system

## Abstract

**Background:**

There is currently no method to predict tyrosine kinase inhibitor (TKI) -induced hepatotoxicity. The purpose of this study was to propose a risk scoring system for hepatotoxicity induced within one year of TKI administration using machine learning methods.

**Methods:**

This retrospective, multi-center study analyzed individual data of patients administered different types of TKIs (crizotinib, erlotinib, gefitinib, imatinib, and lapatinib) selected in five previous studies. The odds ratio and adjusted odds ratio from univariate and multivariate analyses were calculated using a chi-squared test and logistic regression model. Machine learning methods, including five-fold cross-validated multivariate logistic regression, elastic net, and random forest were utilized to predict risk factors for the occurrence of hepatotoxicity. A risk scoring system was developed from the multivariate and machine learning analyses.

**Results:**

Data from 703 patients with grade II or higher hepatotoxicity within one year of TKI administration were evaluated. In a multivariable analysis, male and liver metastasis increased the risk of hepatotoxicity by 1.4-fold and 2.1-fold, respectively. The use of anticancer drugs increased the risk of hepatotoxicity by 6.0-fold. Patients administered H2 blockers or PPIs had a 1.5-fold increased risk of hepatotoxicity. The area under the receiver-operating curve (AUROC) values of machine learning methods ranged between 0.73-0.75. Based on multivariate and machine learning analyses, male (1 point), use of H2 blocker or PPI (1 point), presence of liver metastasis (2 points), and use of anticancer drugs (4 points) were integrated into the risk scoring system. From a training set, patients with 0, 1, 2-3, 4-7 point showed approximately 9.8%, 16.6%, 29.0% and 61.5% of risk of hepatotoxicity, respectively. The AUROC of the scoring system was 0.755 (95% CI, 0.706-0.804).

**Conclusion:**

Our scoring system may be helpful for patient assessment and clinical decisions when administering TKIs included in this study.

## Introduction

Tyrosine kinase inhibitor (TKI) is a prominent cancer treatment. Tyrosine kinase is a major enzyme involved in cell signaling, growth, and division during cell signal transduction (1). TKI inhibits tyrosine kinase, which is involved in cancer (2). Since the U.S. Food and Drug Administration (FDA) approved imatinib for the treatment of chronic myeloid leukemia in 2001, over 30 TKIs have been developed (3, 4).

Hepatotoxicity is a major safety concern when using tyrosine kinase inhibitors (5). The FDA requires five TKIs (lapatinib, pazopanib, ponatinib, regorafenib, and sunitinib) to have black box warnings for liver damage (4, 6). Several studies have investigated TKI-induced hepatotoxicity, mostly in patients experiencing grade I-IV hepatotoxicity (7). However, it is difficult to find clinically significant grade I cases as these include mild and asymptomatic patients.

Since there are no reliable markers for the detection of drug-induced hepatotoxicity, it is important to exclude other possible causes (8, 9). The follow-up period should be limited, as longer observation periods make it difficult to detect drug-induced hepatotoxicity because other factors may come into play (7, 10, 11). The period from TKI initiation to hepatotoxicity onset varies widely, with the latency to the onset of hepatotoxicity reported within two months for crizotinib and several days to several months for lapatinib (12). A proper observation period for hepatotoxicity has not been established, but one year (365 days) may be appropriate.

Machine learning establishes computational modeling for automatic learning based on existing data (13). Since the machine learning approach can devise learning algorithms to deduce clinical action and decision making, it has been applied in various ways in the field of health science, including risk prediction (14, 15). Utilizing various methods of machine learning may build models with higher risk predictability that can explain risk factors.

Risk scoring systems, such as the GerontoNet ADR risk score for elderly patients and TIMI risk score for cardiovascular disease, allow a rapid assessment of patients for medical decision-making and patient management (16). They reveal the relationship between patient risk factors and the incidence of an adverse event and a disease (17). Although it may help clinicians predict hepatotoxicity after TKI administration, a risk scoring system has not yet been investigated.

Although TKI-induced hepatotoxicity is a significant clinical concern, there is currently no tool to predict its development. The purpose of this study is to identify risk factors for TKI-induced hepatotoxicity of grade II or higher that occur within one year of TKI initiation using machine learning methods and to propose a risk scoring system of TKI-induced hepatotoxicity.

## Materials and Methods

### Dataset

The dataset was constructed from five previous studies that demonstrated factors affecting the hepatotoxicity of selected TKIs (gefitinib, erlotinib, crizotinib, imatinib, and lapatinib). The detailed methodology was reported in five published studies. In a gefitinib study, patients with non-small cell lung cancer (NSCLC) were orally administered 250 mg of gefitinib per day (18). Patients with NSCLC or pancreatic cancer were administered 150 mg or 100 mg of erlotinib, respectively (19). Patients with NSCLC containing an anaplastic lymphoma kinase (ALK) rearrangement or c-ros oncogene 1 (ROS1) rearrangement were orally administered 250 mg of crizotinib twice per day (20). Patients with Philadelphia chromosome-positive acute lymphoblastic leukemia (ALL), chronic myeloid leukemia (CML), gastrointestinal stromal tumors (GIST), or other malignancies were orally administered imatinib (100-800 mg/day) (21). Patients with metastatic breast cancer were orally administered lapatinib (750-1250 mg/day) (22). In all five studies, aspartate aminotransferase (AST) and alanine aminotransferase (ALT) levels were measured before initiation of TKI therapy and then every two to three months thereafter. Eligible patients were those who were followed up in a year.

The following baseline data were obtained: sex, age, body weight, height, body surface area (BSA), alcohol history, underlying disease, liver metastasis, HBsAg, and concomitant medications. Concomitant drugs included cytochrome P450 (CYP) 3A4 inducers, CYP3A4 inhibitors, anticancer drugs, H2 blockers, and proton pump inhibitors (PPIs). CYP3A4 inducers included bosentan, carbamazepine, dexamethasone, efavirenz, ethosuximide, etravirine, fosphenytoin, modafinil, nafcillin, oxcarbazepine, phenobarbital, phenytoin, prednisolone, primidone, rifabutin, and rifampicin (rifampin). CYP3A4 inhibitors were amiodarone, aprepitant, atazanavir, cimetidine, ciprofloxacin, clarithromycin, cyclosporine, danazol, diltiazem, erythromycin, fluconazole, fluoxetine, fluvoxamine, grapefruit juice, itraconazole, ketoconazole, nicardipine, nifedipine, posaconazole, ritonavir, tamoxifen, verapamil, and voriconazole. Anticancer drugs included capecitabine, cisplatin, cyclophosphamide, cytarabine, docetaxel, trastuzumab, vincristine, and vinorelbine. H2 blockers were cimetidine, famotidine, lafutidine, nizatidine, and ranitidine. PPIs included (es)omeprazole, (dex)lansoprazole, pantoprazole, and rabeprazole.

### Assessment of Hepatotoxicity

Serum AST and ALT values were assessed according to the severity of hepatotoxicity. The hepatotoxicity grade was determined using Common Terminology Criteria for Adverse Events (CTCAE) version 4.0. The CTCAE defines grade I, grade II, grade III, and grade IV toxicity levels of AST and ALT as 1-3 times, 3-5 times, 5-20 times, and more than 20 times the upper limit of normal, respectively. In this study, hepatotoxicity was defined as grade II or higher.

### Statistical Analysis

The chi-squared or Fisher’s exact test was performed to compare categorical variables between patients with and without hepatotoxicity. Multivariate logistic regression analysis was performed to identify independent risk factors for hepatotoxicity. Factors having a P-value < 0.05 from the univariate analysis with strong confounding factors (age, BSA, and sex) were included in the multivariate analysis. The odds ratio (OR) and adjusted OR were calculated by univariate and multivariate analyses, respectively.

Machine learning models were developed to predict the risk factors for hepatotoxicity. Classification methods, such as five-fold cross-validated multivariable logistic regression, elastic net, and random forest (RF) were utilized with an R package caret. For cross-validation, the dataset was randomly split into five equal folds. After portioning one data sample into five subsets, four subsets were used to construct machine learning models and the other subset was used for model validation. Each cross-validation iteration was repeated 100 times. The area under the receiver-operating curve (AUROC) was developed to predict hepatotoxicity.

A risk scoring system was developed from the multivariate and machine learning analyses. We randomly divided the data by a ratio of 7:3. Among a total of 703 samples included in this study, data from 503 patients were used to construct a risk scoring system, and the other 200 data were used to validate it. For the risk score, each coefficient from the logistic regression model was divided by the smallest one and rounded to the nearest integer.

P-values less than 0.05 were considered statistically significant. Univariate and multivariate analyses were performed with the Statistical Package for Social Sciences (SPSS) version 20.0 for Windows (SPSS Inc., Chicago, Illinois, USA). Machine learning models were developed using R software version 3.6.0 (RFoundation for Statistical Computing, Vienna, Austria).

## Results

Among the 999 patients eligible in this study, patients were excluded if they did not have AST/ALT value results before TKI administration (n = 72), if they had elevated AST/ALT before TKI administration (n = 123), and if they already had underlying liver disease (n = 101). We analyzed data from 703 patients. For the excluded patients, the mean age, proportion of patients ≥ 60 years, and proportion of males were 60.6 ± 13.1 years, 56.3%, and 47.8%, respectively. There were no significant differences in the mean age or proportion of sex between the included and excluded patients.

As shown in **Table 1**, 191 patients experienced the hepatotoxicity induced by the selected TKIs during the study period. Around half (50.2%) of the patients were older than 60 years of age. Drugs concomitantly administered with TKI included CYP3A4 inhibitors (n = 26), CYP3A4 inducers (n = 33), H2 blockers (n = 202), PPIs (n = 114), and anticancer drugs (n = 161). In the univariate analysis, liver metastasis, CYP3A4 inhibitors, CYP3A4 inducers, anticancer drugs, H2 blockers, PPIs, and H2 blockers or PPIs were significant factors for hepatotoxicity.

**Table 1:** Hepatotoxicity of TKI administration.

Characteristics		No. (%)(n=703)	Hepatotoxicity, No (%)		*P*-value
			Absence	Presence	
			(n=512)	(n=191)	
Sex					0.980
	Female	408 (58.0)	297 (58.0)	111 (58.1)	
	Male	295 (42.0)	215 (42.0)	80 (41.9)	
Age, years					0.064
	<60	350 (49.8)	244 (47.7)	106 (55.5)	
	≥60	353 (50.2)	268 (52.3)	85 (44.5)	
BW, kg^a^					0.253
	<60	379 (54.6)	268 (53.3)	111 (58.1)	
	≥60	315 (45.4)	235 (46.7)	80 (41.9)	
Height, cm^b^					0.540
	<160	336 (48.5)	247 (49.2)	89 (46.6)	
	≥160	357 (51.5)	255 (50.8)	102 (53.4)	
BSA, m^2c^					0.346
	<1.6	321 (46.3)	227 (45.2)	94 (49.2)	
	≥1.6	372 (53.7)	275 (54.8)	97 (50.8)	
Alcohol history^d^					0.257
	Yes	86 (27.7)	67 (29.4)	19 (22.9)	
	No	225 (72.3)	161 (70.6)	64 (77.1)	
CVD or DM					0.289
	Yes	254 (36.1)	191 (37.3)	63 (33.0)	
	No	449 (63.9)	321 (62.7)	128 (67.0)	
Liver metastasis					<0. 001
	Yes	76 (10.8)	34 (6.6)	42 (22.0)	
	No	627 (89.2)	478 (93.4)	149 (78.0)	
HBsAg^e^					0.556
	Yes	18 (2.6)	12 (2.4)	6 (3.2)	
	No	665 (97.4)	485 (97.6)	180 (96.8)	
CYP3A4 inhibitors					<0. 001
	Yes	26 (3.7)	11 (2.1)	15 (7.9)	
	No	677 (96.3)	501 (97.9)	176 (92.1)	
CYP3A4 inducers					<0. 001
	Yes	33 (4.7)	14 (2.7)	19 (9.9)	
	No	670 (95.3)	498 (97.3)	172 (90.1)	
H2 blockers					0.005
	Yes	202 (28.7)	132 (25.8)	70 (36.6)	
	No	501 (71.3)	380 (74.2)	121 (63.4)	
PPIs					0.021
	Yes	114 (16.2)	73 (14.3)	41 (21.5)	
	No	589 (83.8)	439 (85.7)	150 (78.5)	
H2 blockers/PPIs					<0. 001
	Yes	281 (40.0)	183 (35.7)	98 (51.3)	
	No	422 (60.0)	329 (64.3)	93 (48.7)	
Anticancer drugs					<0. 001
	Yes	161 (22.9)	63 (12.3)	98 (51.3)	
	No	542 (77.1)	449 (87.7)	93 (48.7)	

BW, body weight; BSA, body surface area; CVD, cardiovascular diseases; CYP3A4, cytochrome P450 3A4; DM, diabetes mellitus; PPI, proton pump inhibitor.

a Body weight data for 9 patients were missing.

b Height data for 10 patients were missing.

c Body surface area data for 10 patients were missing.

d Alcohol history data for 392 patients were missing.

e HBsAg data for 20 patients were missing.

Multivariate analysis demonstrated that male patients and patients with liver metastasis had increased risk for TKI-induced hepatotoxicity by 1.4-fold and 2.1-fold, respectively. The use of anticancer drugs increased the risk of hepatotoxicity by 6.0-fold. Patients using H2 blockers or PPIs had a 1.5-fold increased risk of hepatotoxicity (**Table 2**).

**Table 2:** Univariate and multivariate analyses to identify predictors for hepatotoxicity related to TKI administration.

Characteristics	Unadjusted OR	Adjusted OR
	(95% CI)	(95% CI)
Male	0.996 (0.711-1.394)	1.418 (0.962-2.090)
Age ≥ 60 years	0.730 (0.523-1.020)	
BSA ≥ 1.6	0.852 (0.610-1.189)	
Liver metastasis	3.963 (2.432-6.457)^**^	2.146 (1.224-3.762)^**^
CYP3A4 inhibitors	3.882 (1.750-8.611)^**^	
CYP3A4 inducers	3.929 (1.928-8.007)^**^	
Anticancer drugs	7.510 (5.098-11.063)^**^	6.002 (3.956-9.107)^**^
H2 blockers	1.665 (1.168-2.375)^**^	
PPIs	1.644 (1.075-2.514)^*^	
H2 blockers/PPIs	1.894 (1.353-2.652)^**^	1.461 (0.987-2.163)

BSA, body surface area; CYP3A4, cytochrome P450 3A4; OR, odds ratio; PPI, proton pump inhibitor.

*P < 0.05, **P < 0.01.

**Figure 1:**
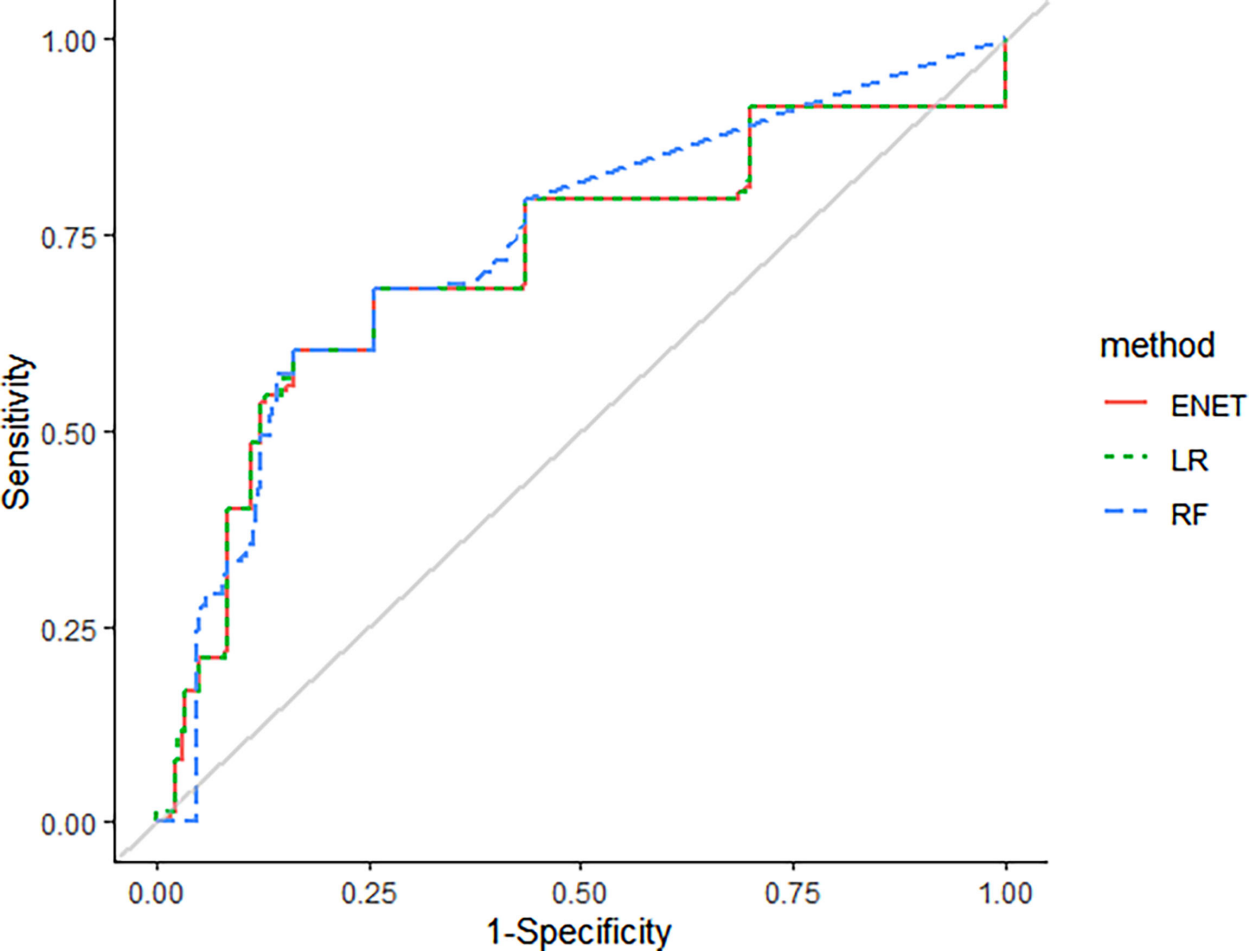
The receiver-operating curves (ROC) for five-fold cross-validated multivariate logistic regression, elastic net, and random forest (RF).

Machine learning methods were utilized to construct a prediction model for TKI-associated hepatotoxicity. The AUROC values (mean, 95% CI) across 100 random iterations using five-fold cross-validated multivariate logistic regression, elastic net, and RF models were 0.75, 0.75, and 0.73, respectively (**Table 3**). The ROC for five-fold cross-validated multivariate logistic regression, elastic net, and RF are shown in **Figure 1**. The hyperparameters and R code that we used are shown in **Table 4** and **Supplementary File 1**, respectively.

**Table 3:** Machine learning models’ performance.

Model	AUROC (95% CI)	Sensitivity	Specificity
Multivariate logistic regression	0.75 (0.701 - 0.804)	0.601	0.836
Elastic net	0.75 (0.703 - 0.805)	0.601	0.838
Random forest	0.73 (0.681 - 0.775)	0.601	0.838

AUROC, area under the receiver-operating curve; CI, confidence interval.

**Table 4:** Machine learning model specifics.

Method	Hyperparameter	
	Model specification and search grids	Selected values
Elastic net	λ: 100 equally spaced values in logarithmic scale between 10^-4^ and 0	λ: 0.03511192
	α: 0, 0.2, 0.4, 0.6, 0.8, 1	α: 0
Random forests	mtry: 1-4	mtry: 1

SVM, Support vector machine.

**Figure 2:**
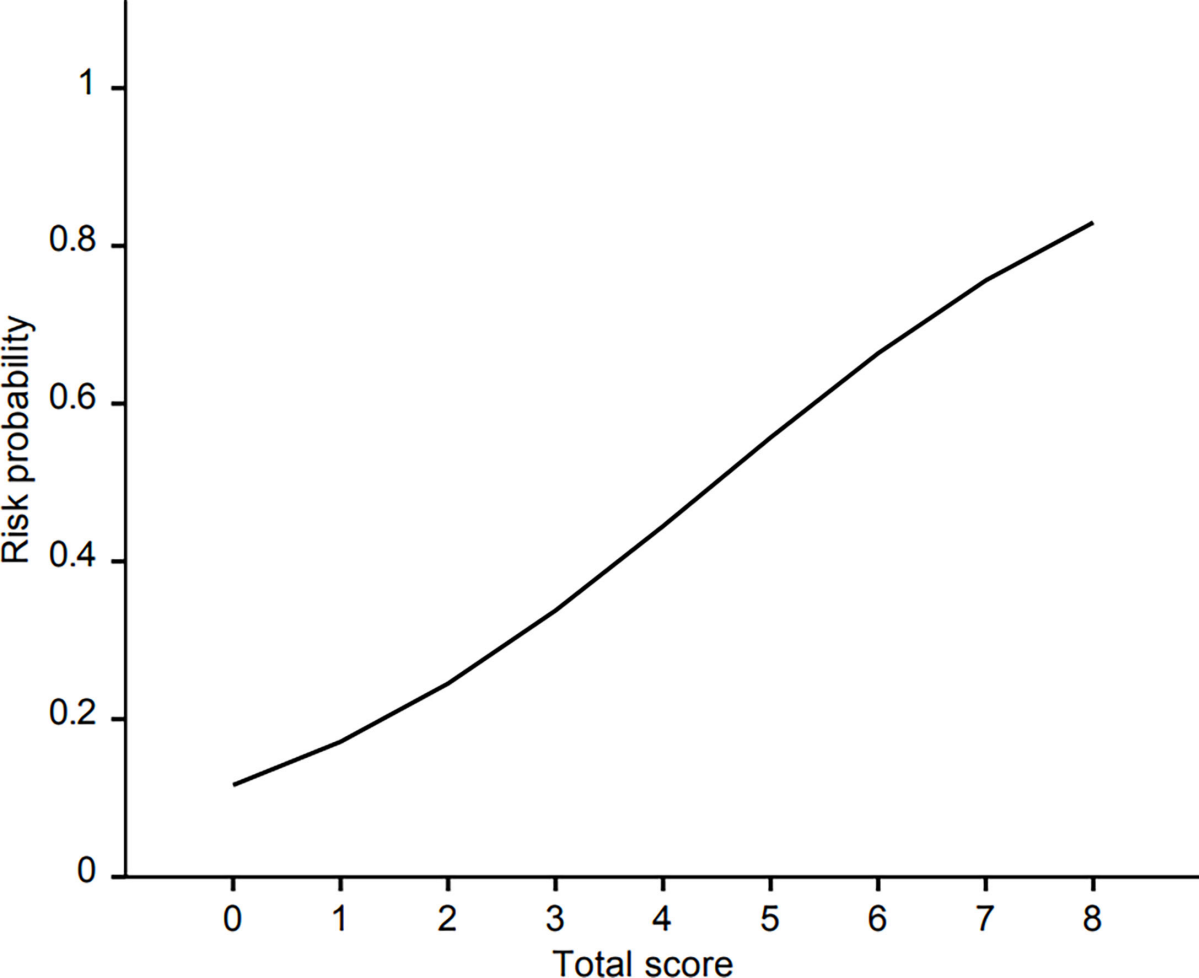
The logistic regression curve of the probability of hepatotoxicity versus the proposed scoring scale.

For the construction of risk scoring system, male (1 point), use of H2 blockers or PPIs (1 point), presence of liver metastasis (2 points), and use of anticancer drugs (4 points) were integrated into the analysis. From a training set, patients with 0, 1, 2-3, and 4-7 points showed approximately 9.8%, 16.6%, 29.0%, and 61.5% risk of hepatotoxicity, respectively. The respective value of the validation set was 10.2, 19.3, 30.8, and 57.1%. Although there were only two patients who scored 8 points (100% risk), they were all included in the training set. The logistic regression curve by mapping the scores to risk scores is presented in **Figure 2**, and the risk probability according to scores using logistic regression is shown in **Table 5**. The AUROC of the scoring system was 0.755 (95% CI 0.706-0.804).

**Table 5:** Risk of hepatotoxicity according to scores using logistic regression.

Score	0	1	2	3	4	5	6	7	8
Risk probability	0.116	0.172	0.245	0.338	0.445	0.557	0.664	0.756	0.830

## Discussion

This study demonstrated that the use of H2 blockers or PPIs and anticancer drugs increased the risk of the hepatotoxicity induced by the TKIs selected in this study (crizotinib, erlotinib, gefitinib, imatinib, and lapatinib) by 1.5-fold and 6.0-fold, respectively. Patients with liver metastasis and male patients had an increased risk of TKI-induced hepatotoxicity by 2.1-fold and by 1.4-fold, respectively. Machine learning analyses indicated good performance (higher than 0.7) of the constructed model.

In our study, the presence of liver metastasis was a significant factor with the two-fold increase in hepatotoxicity by TKIs included in this study. Because patients with elevated AST and ALT were excluded, all patients had normal AST/ALT values at the start of the study. The relationship between liver metastasis and drug-induced hepatotoxicity has been rarely reported. However, a retrospective observational study of pembrolizumab-induced liver injury showed that patients with pre-existing liver metastasis were at a 3.6-fold higher risk of developing hepatotoxicity compared to patients with no liver metastasis (23). As the main metabolic site for most TKIs is the liver, the presence of liver metastasis may lead to asymptomatic liver damage before TKI use and may amplify the effects of TKI-induced hepatotoxicity.

TKIs are often used in combination with other anticancer drugs. Previous studies have reported hepatotoxicity by many anticancer drugs, including methotrexate, cisplatin, gemcitabine, and paclitaxel (24). Thus, anticancer drugs used in combination with TKIs not only affect hepatotoxicity by themselves but may further aggravate the severity of hepatotoxicity caused by TKIs.

For the construction of the risk scoring system, we included all factors that remained in the final multivariate analysis model, regardless of statistical significance. In addition to liver metastasis and anticancer drugs, male and the use of H2 blockers/PPIs were included in the risk scoring system. Contrary to our expectations, male sex increased the risk of TKI-induced hepatotoxicity in our study. Several studies have demonstrated that female patients generally had a higher risk of adverse drug reactions compared to male patients, and these results were similar for drug-induced hepatotoxicity (25, 26). Physiological or biological differences which can affect drug toxicity may contribute to these gender differences (27). Our unexpected result is probably due to the effect of alcohol history. Male patients accounted for the majority (70%) of patients with a history of alcohol use, and 70% of these individuals had hepatotoxicity. Considering that female patients accounted for more than half of our study population, alcohol history may be an influencing factor in the higher incidence of hepatotoxicity in male patients.

Concomitant use of PPIs or H2 blockers increased the risk of hepatotoxicity compared to non-users. ATP-binding cassette superfamily G member 2 (ABCG2) and ATP-binding cassette subfamily B member 1 (ABCB1) are drug efflux transporters situated in the liver (28). Since PPIs are known as an ABCG2 inhibitors, concomitant use of ABCG2 substrates and PPIs can increase the blood concentration of drugs that are ABCG2 substrates (18, 19). Among the five drugs included in our study, gefitinib and erlotinib are substrates of ABCG2. Since half of the total study population was patients administered these drugs, this may have affected the analysis of PPIs as a hepatotoxicity factor.

Both H2 blockers and TKIs are ABCB1 substrates. Co-administration of both ABCB1 substrates can cause competitive efflux transport, meaning other ABCB1 substrates such as TKIs remain in the liver instead of H2 blockers exiting. This increases the risk of TKI-induced hepatotoxicity.

TKIs as a class with different mechanisms were included in this study. Like differences between epidermal growth factor receptor (EGFR) TKIs and non-receptor TKIs, differences in mechanisms may affect the occurrence of TKI-induced toxicity (29). However, this was not found in this study, probably because many TKIs have multiple targets, as imatinib mainly targets bcr-abl but also affects a receptor tyrosine kinase, platelet-derived growth factor receptor (PDGFR).

TKIs included in this study were used as a single daily dose (gefitinib 250 mg, crizotinib 250 mg, and lapatinib 1250 mg) except for imatinib and erlotinib, and the effects of drug doses on hepatotoxicity were not found in both drugs. In the case of imatinib, the dose range was 100 to 800 mg daily; it was not a significant factor for imatinib-induced hepatotoxicity in the multivariate analysis. For erlotinib, the daily dose was either 100 mg or 150 mg, and the statistical significance was not found. Since the three drugs among five TKIs in this study were used as a single dose, the effect of drug doses on clinical efficacy and safety should be further investigated.

The AUROC values of machine learning methods ranged between 0.73-0.75. The machine learning methods that showed the best AUROC values were the five-fold multivariable logistic regression model and the elastic net model, a penalized linear regression model that combined the penalties of the lasso and ridge methods (30). The constructed risk scoring system showed good performance with the AUROC value of 0.75.

There are several limitations to this study. The main limitation is the retrospective design of our study. It was impossible to obtain the patient’s drug concentration to assess the relationship with the onset of hepatotoxicity or the patient’s tissue to analyze the pattern of hepatotoxicity. In addition, not all TKIs were included in this study; especially, only one TKI among five TKIs with black box warning for hepatotoxicity was analyzed, Therefore, it needs to be cautious to apply this result to other TKIs. Since a relatively large number of patients were excluded according to the exclusion criteria, it is possible that real-world data could be different. However, the characteristics between included patients and excluded patients were not significantly different. Despite several shortcomings, our study is significant because it is the first to develop a risk scoring system for the hepatotoxicity caused by the selected TKIs in cancer patients. Furthermore, machine learning models were used to predict the increased risk of hepatotoxicity.

In conclusion, our study demonstrated that the presence of liver metastasis and the concurrent use of PPIs or H2 blockers were related to TKI-induced hepatotoxicity. Male patients and patients administered anticancer drugs experienced an increased risk of hepatotoxicity. Before applying these results to clinical settings, it is necessary to consider other factors that may affect the efficacy and safety of the TKIs, such as daily dose, drug interaction, and genetic factors. Considering our retrospective study design and only five selected TKIs were included in this study, further prospective studies are needed to validate our findings.

## Data Availability Statement

The raw data supporting the conclusions of this article will be made available by the authors, without undue reservation.

## Ethics Statement

All procedures performed in five studies involving human participants were in accordance with the ethical standards of the relevant ethics committees, which approved the studies. The Ethics committees were as follow; the Clinical Research Ethics Committee of the Seoul National University Hospitals, The Asan Medical Center Clinical Research Ethics Committee, the Institutional Review Board of National Cancer Center, Korea. Written informed consent for participation was not required for this study due to the retrospective nature of this study.

## Author Contributions

All the authors have made substantial contributions to the conception of the study. All the authors contributed to designing the study. JH, SC, MK, JM, DJ, and JK contributed to material preparation and data collection. JH, JY, and HG performed data analysis and interpretation. JH contributed to drafting of the manuscript. HG contributed to critical revision of the manuscript. All authors approved the final manuscript.

## Conflict of Interest

The authors declare that the research was conducted in the absence of any commercial or financial relationships that could be construed as a potential conflict of interest.

## Publisher’s Note

All claims expressed in this article are solely those of the authors and do not necessarily represent those of their affiliated organizations, or those of the publisher, the editors and the reviewers. Any product that may be evaluated in this article, or claim that may be made by its manufacturer, is not guaranteed or endorsed by the publisher.
