# Supplementary material for: A Risk Scoring System Utilizing Machine Learning Methods for Hepatotoxicity Prediction One Year After the Initiation of Tyrosine Kinase Inhibitors
Source: Front Oncol. 2022 Mar 8;12:790343. doi: 10.3389/fonc.2022.790343 (PMC8957909; doi:10.3389/fonc.2022.790343)
Supplement: Supplementary file 1 [file Table_1.docx]

library(caret)

library (ROCR)

library (ggplot2)

raw.data <- read.csv("FILE", stringsAsFactors = F)

fun.ci.95 <- function(x){

# aaa <-signif(quantile(x, probs = c(0.025, 0.975)),3)

temp.mean <- mean(x)

temp.var <- sum((x - temp.mean)^2)/(500-1)

temp.t.0.025 <- qt(c(.025, .975), df=499)

aaa <- signif(temp.mean + temp.t.0.025* sqrt((1/500+ 1/4)*temp.var),3)

print(paste0("[", aaa[1], " - ", aaa[2], "]"))

}

#glm

train_control <- trainControl(method="repeatedcv",

number=5, repeats=100,

savePredictions = TRUE,

classProbs = TRUE,

summaryFunction=twoClassSummary)

idx.na <- apply(raw.data,1, function(x){

any(is.na(x))

})

output <- ifelse(raw.data$Outcome == 0, "no", "yes")

output <- factor(output,levels = c("no", "yes"))

set.seed(12345)

glm_grid <- train(x = raw.data[!idx.na,-1], y =output[!idx.na], method = "glm",

family = binomial,

trControl=train_control,

#preProcess = c("center", "scale"),

metric = "ROC")#,

#tuneGrid = grid)

glm_grid

glm_grid$bestTune

mean(glm_grid$resample[,1])

fun.ci.95(glm_grid$resample[,1])

plot(glm_grid)

glm_grid$results

temp.cv.folds <- unique(glm_grid$pred$Resample)

glm.aupr <- rep(0, length(temp.cv.folds))

i = 0

for(idx.fold in temp.cv.folds){

i <- i+1

temp.obs <- glm_grid$pred$obs[glm_grid$pred$Resample == idx.fold]

temp.pred <- glm_grid$pred$yes[glm_grid$pred$Resample == idx.fold]

confusionMatrix(glm_grid$pred$pred[1:35], glm_grid$pred$obs[1:35],positive = "yes")

cv.pred.glm_grid <- aggregate(yes~rowIndex,glm_grid$pred,mean)

cut.offs <- c(0,cv.pred.glm_grid$yes[order(cv.pred.glm_grid$yes)])

#idx.cut.offs <- 1

glm.accuracy <- data.frame(cut.offs =cut.offs,

F1 = NA,

Bal.Accu = NA)

for(idx.cut.offs in 1:length(cut.offs)){

temp.pred.class <- ifelse(cut.offs[idx.cut.offs] < cv.pred.glm_grid$yes, "yes", "no")

temp.pred.class <- factor(temp.pred.class,levels = c("no", "yes"), labels = c("no","yes"))

temp.confusion <- confusionMatrix(temp.pred.class, factor(output[!idx.na]),positive = "yes")

glm.accuracy[idx.cut.offs,"F1"] <- temp.confusion$byClass["F1"]

glm.accuracy[idx.cut.offs,"Bal.Accu"]<- temp.confusion$byClass["Balanced Accuracy"]

}

with(glm.accuracy, plot(cut.offs, F1))

with(glm.accuracy, plot(cut.offs, Bal.Accu))

glm.accuracy$cut.offs[which.max(glm.accuracy$F1)]

glm.accuracy$cut.offs[which.max(glm.accuracy$Bal.Accu)]

cut.off.selected <- VALUE

confusionMatrix(factor(ifelse(cv.pred.glm_grid$yes > cut.off.selected, "yes", "no")),

factor(output[!idx.na]),positive = "yes")

#elastic net

grid <- expand.grid( .alpha = seq(0,1,length.out = 6),

.lambda = 10^(seq(-4,0,length.out = 100)))

head(grid)

train_control <- trainControl(method="repeatedcv",

number=5, repeats=100,

savePredictions = TRUE,

classProbs = TRUE,

summaryFunction=twoClassSummary)

idx.na <- apply(raw.data,1, function(x){

any(is.na(x))

})

output <- ifelse(raw.data$Outcome == 0, "no", "yes")

output <- factor(output,levels = c("no", "yes"))

set.seed(12345)

enet_grid <- train(x = raw.data[!idx.na,-1], y =output[!idx.na], method = "glmnet",

#family = binomial,

trControl=train_control,

#preProcess = c("center", "scale"),

metric = "ROC",

tuneGrid = grid)#,

enet_grid$finalModel$beta[,12]

enet_grid$bestTune

mean(enet_grid$resample[,1])

fun.ci.95(enet_grid$resample[,1])

plot(enet_grid)

summary(enet_grid)

enet_grid$results

temp.idx.best <- enet_grid$pred$alpha ==enet_grid$bestTune[1,1]& enet_grid$pred$lambda == enet_grid$bestTune[1,2]

temp.pred.bestTune <- enet_grid$pred[temp.idx.best,]

temp.cv.folds <- unique(temp.pred.bestTune$Resample)

enet.aupr <- rep(0, length(temp.cv.folds))

i = 0

for(idx.fold in temp.cv.folds){

i <- i+1

temp.obs <- temp.pred.bestTune$obs[temp.pred.bestTune$Resample == idx.fold]

temp.pred <- temp.pred.bestTune$yes[temp.pred.bestTune$Resample == idx.fold]

temp.idx.best <- enet_grid$pred$alpha ==enet_grid$bestTune[1,1]& enet_grid$pred$lambda == enet_grid$bestTune[1,2]

temp.pred.bestTune <- enet_grid$pred[temp.idx.best,]

cv.pred.enet_grid <- aggregate(yes~rowIndex,temp.pred.bestTune,mean)

cut.offs <- c(0, cv.pred.enet_grid$yes[order(cv.pred.enet_grid$yes)])

#idx.cut.offs <- 1

enet.accuracy <- data.frame(cut.offs =cut.offs,

F1 = NA,

Bal.Accu = NA)

for(idx.cut.offs in 1:length(cut.offs)){

temp.pred.class <- ifelse(cut.offs[idx.cut.offs] < cv.pred.enet_grid$yes, "yes", "no")

temp.pred.class <- factor(temp.pred.class,levels = c("no", "yes"), labels = c("no","yes"))

temp.confusion <- confusionMatrix(temp.pred.class, factor(output[!idx.na]),positive = "yes")

enet.accuracy[idx.cut.offs,"F1"] <- temp.confusion$byClass["F1"]

enet.accuracy[idx.cut.offs,"Bal.Accu"]<- temp.confusion$byClass["Balanced Accuracy"]

}

with(enet.accuracy, plot(cut.offs, F1))

with(enet.accuracy, plot(cut.offs, Bal.Accu))

enet.accuracy$cut.offs[which.max(enet.accuracy$F1)]

enet.accuracy$cut.offs[which.max(enet.accuracy$Bal.Accu)]

cut.off.selected <- VALUE

confusionMatrix(factor(ifelse(cv.pred.enet_grid$yes > cut.off.selected, "yes", "no")),

factor(output[!idx.na]),positive = "yes")

#RF caret

grid <- expand.grid(.mtry = c(1:4) )

train_control <- trainControl(method="repeatedcv",

number=5, repeats=100,

savePredictions = TRUE,

classProbs = TRUE,

summaryFunction=twoClassSummary)

idx.na <- apply(raw.data,1, function(x){

any(is.na(x))

})

output <- ifelse(raw.data$Outcome == 0, "no", "yes")

set.seed(12345)

rf_grid <- train(x = raw.data[!idx.na,-1], y = output[!idx.na], method = "rf",

trControl=train_control,

#preProcess = c("center", "scale"),

metric = "ROC",

tuneGrid = grid)

rf_grid

rf_grid$bestTune

mean(rf_grid$resample[,1])

fun.ci.95(rf_grid$resample[,1])

plot(rf_grid)

rf_grid$results

temp.idx.best <- rf_grid$pred$mtry ==rf_grid$bestTune[1,1]

temp.pred.bestTune <- rf_grid$pred[temp.idx.best,]

temp.cv.folds <- unique(temp.pred.bestTune$Resample)

rf.aupr <- rep(0, length(temp.cv.folds))

i = 0

for(idx.fold in temp.cv.folds){

i <- i+1

temp.obs <- temp.pred.bestTune$obs[temp.pred.bestTune$Resample == idx.fold]

temp.pred <- temp.pred.bestTune$yes[temp.pred.bestTune$Resample == idx.fold]

pred.obj <- prediction(temp.pred, temp.obs)

temp.idx.best <- rf_grid$pred$mtry ==rf_grid$bestTune[1,1]

temp.pred.bestTune <- rf_grid$pred[temp.idx.best,]

cv.pred.rf_grid <- aggregate(yes~rowIndex,temp.pred.bestTune,mean)

cut.offs <- c(0, cv.pred.rf_grid$yes[order(cv.pred.rf_grid$yes)])

#idx.cut.offs <- 1

rf.accuracy <- data.frame(cut.offs =cut.offs,

F1 = NA,

Bal.Accu = NA)

for(idx.cut.offs in 1:length(cut.offs)){

temp.pred.class <- ifelse(cut.offs[idx.cut.offs] < cv.pred.rf_grid$yes, "yes", "no")

temp.pred.class <- factor(temp.pred.class,levels = c("no", "yes"), labels = c("no","yes"))

temp.confusion <- confusionMatrix(temp.pred.class, factor(output[!idx.na]),positive = "yes")

rf.accuracy[idx.cut.offs,"F1"] <- temp.confusion$byClass["F1"]

rf.accuracy[idx.cut.offs,"Bal.Accu"]<- temp.confusion$byClass["Balanced Accuracy"]

}

with(rf.accuracy, plot(cut.offs, F1))

with(rf.accuracy, plot(cut.offs, Bal.Accu))

rf.accuracy$cut.offs[which.max(rf.accuracy$F1)]

rf.accuracy$cut.offs[which.max(rf.accuracy$Bal.Accu)]

cut.off.selected <- VALUE

confusionMatrix(factor(ifelse(cv.pred.rf_grid$yes > cut.off.selected, "yes", "no")),

factor(output[!idx.na]),positive = "yes")
